# Changes in perceived neighborhood social cohesion and self-assessed health: 17-year follow-up of the Dutch GLOBE study

**DOI:** 10.1093/eurpub/ckae168

**Published:** 2024-11-01

**Authors:** Vernon Cail, Joost Oude Groeniger, Mariëlle A Beenackers, Frank J van Lenthe

**Affiliations:** Department of Public Health, Erasmus MC University Medical Center Rotterdam, Rotterdam, The Netherlands; Department of Public Health, Erasmus MC University Medical Center Rotterdam, Rotterdam, The Netherlands; Department of Public Health, Erasmus MC University Medical Center Rotterdam, Rotterdam, The Netherlands; Department of Public Health, Erasmus MC University Medical Center Rotterdam, Rotterdam, The Netherlands

## Abstract

Prior research has indicated that residents who perceive their neighborhood as more cohesive have better mental and physical health than those with lower perceived neighborhood social cohesion. However, because most studies are based on cross-sectional data, it remains unclear whether improving the perceptions of social cohesion leads to better health over time. This study applied random effects within-between models to examine the within-individual and between-individual associations of perceived neighborhood social cohesion and poor self-assessed health (SAH) in a cohort of Dutch adults with 17-year follow-up. We also tested whether such associations varied by age, educational level, and gender. The results of pooled analyses indicated that higher perceived neighborhood social cohesion was associated with better SAH [odds ratio (OR): 0.72; 95% confidence interval (CI): 0.65, 0.80], but did not find conclusive evidence that within-individual changes in perceived neighborhood social cohesion were associated with SAH (OR: 0.96; 95% CI: 0.89, 1.04). We also did not observe any moderating effects for age, educational level, or gender. This study provides some evidence that improving social cohesion in neighborhoods may be a beneficial health promotion strategy.

## Introduction

Public health officials and policymakers face persistent challenges when addressing neighborhood inequalities in health [1]. In order to advance the development of interventions designed to improve health in neighborhoods, it is important to know how residents’ perceptions of their neighborhood impacts their health [2]. One determinant that is widely examined in this context is perceived neighborhood social cohesion [3, 4]. Within the field of public health, social cohesion is often considered a group-level construct that refers to the “extent of connectedness and solidarity among groups in a society” [5]. It is believed that higher perceptions of neighborhood cohesion are beneficial to individual health through various mechanisms [6]. The first of these is collective efficacy, which refers to residents’ perceived ability to undertake collectively desired actions [7]. Collective efficacy has been shown to protect communities against neighborhood violence, which is associated with adverse health outcomes [2]. The second mechanism, informal social control, characterizes the neighborhood’s ability to enforce and maintain social norms, which can inhibit deviant health behaviors such as underage drinking and smoking [3]. Finally, reciprocity among neighborhood residents involves residents helping each other with the expectation that the favor will be returned [3]. This mechanism helps ensure that health-promoting resources are distributed throughout the community. Despite its potential for improving health, social cohesion also has the potential to harm groups of people [8]. For example, tightly knit communities have the ability to exclude minorities or restrict individual freedoms [8].

There is a burgeoning body of empirical research examining the effects of neighborhood social cohesion on a wide range of health outcomes, especially self-assessed health (SAH) [4]. SAH is a widely used measure of individual health, because it can be easily administrated and has been shown to be a strong predictor of mortality [9–11] and objective health outcomes [12]. A 2019 review of the literature found considerable evidence supporting the association between social cohesion and SAH [4]. It should be noted, however, that most of these findings were largely based on cross-sectional data. Therefore, they lack the ability to draw causal conclusions and to determine the temporal direction, leaving doubt as to whether improving the perceptions of social cohesion will actually lead to better health.

In response to these limitations, there is a call to use longitudinal study designs and advanced statistical methods for approaching causal relationships [4]. One method that is proliferating in epidemiologic research is fixed-effects models [13]. When applied to longitudinal data, fixed-effects models can estimate associations between changes in exposures and outcomes within individuals. The primary appeal to using these models is that individuals act as their own control by comparing measures at different time points, thereby removing confounding from unobserved time-invariant factors [13]. The drawback to fixed-effects models is that the effects of time-invariant covariates (e.g. gender) cannot be estimated [13]. An even more flexible model is the random-effects within-between (REWB) model, which has the ability to estimate both within-individual and between-individual associations simultaneously [14, 15].

Previous research also suggests that the relationship between the social environment and health may vary by certain sociodemographic factors [16–18]. Older adults, especially those with limited mobility, rely more on their own social network [19], spend more time in their neighborhood, and stay longer in the same living environment than younger adults [20]. Consequently, the local community might be more important for the health of older individuals than younger adults. Differences across socioeconomic positions (SEP) may also modify the relationship between neighborhood social cohesion and health. The level of educational attainment, a common SEP indicator, is a strong predictor of where individuals will reside and who they interact with. Less-educated individuals generally have less access to resources and more exposure to neighborhood disorder compared to more-educated individuals [21]. Some research suggest that improvements in social cohesion could buffer adverse exposures for people with low SEP [17]. Therefore, people with less education may have a greater benefit in health from improvements in social cohesion than more-educated people [17]. Finally, while several studies investigated gender differences in the strength of the association between neighborhood factors and health, evidence is contradictory. For example, a stronger association has been reported between perceptions of neighborhood social cohesion and mental health in men than in women [22]. Yet, stronger associations between related environmental factors (such as trust) and SAH for women compared to men have been found as well [16]. While this illustrates the importance of more research, it leaves us agnostic about the direction of the gender differences.

To fill these important knowledge and methodological gaps, the current study aims to examine the within-individual and between-individual associations of perceived neighborhood social cohesion and SAH. We hypothesize that (i) higher perceived social cohesion is positively associated with better SAH (between-individual association), and (ii) changes in perceived neighborhood social cohesion are positively associated with changes in SAH (within-individual association). The second aim of this analysis is to determine whether the relationship between perceived neighborhood social cohesion and health varies across age, SEP, and gender. We hypothesize that the between-individual and within-individual association of higher perceived social cohesion with better SAH will be larger among older adults compared to young adults, and individuals with less education compared to individuals with more education. Given the contradictory evidence for gender, we do not hypothesize a direction for this analysis.

## Methods

### Data

This study used data from the GLOBE study, a prospective cohort study aimed at understanding the socioeconomic inequalities in health in the Netherlands. Baseline measurements were taken in 1991 on a representative sample of 27 070 adults from the city of Eindhoven and surrounding villages [23]. A smaller subset was followed up in subsequent waves via postal surveys and oral interviews [23]. More details about the GLOBE study are available elsewhere [23]. The use of personal data in the GLOBE study is in compliance with the Dutch Personal Data Protection Act and the Municipal Database Act and has been registered with the Dutch Data Protection Authority (number 1248943).

**Figure 1.**
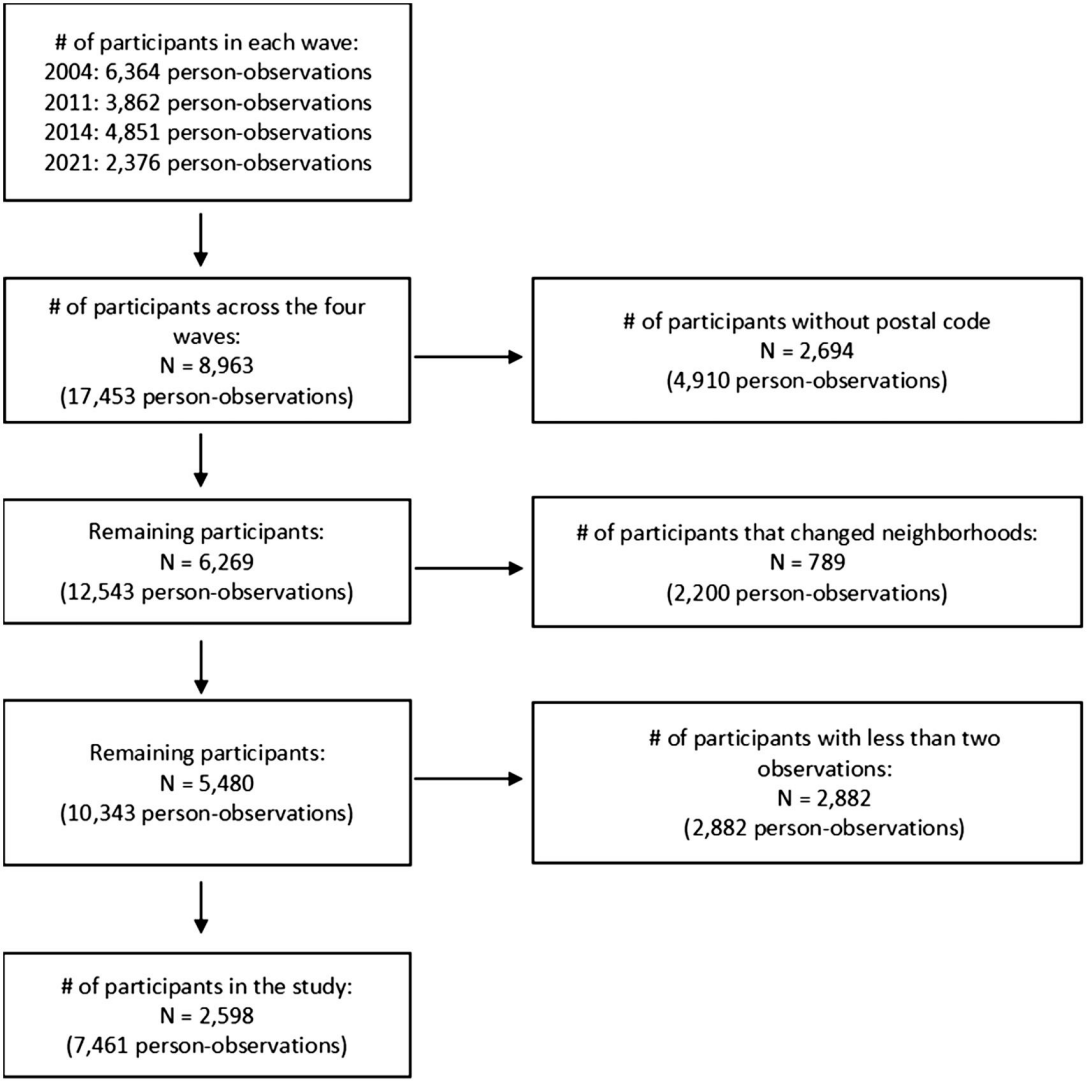
Flow chart of the participants in the study.

This analysis included data from the follow-up surveys in 2004, 2011, 2014, and 2021. We were primarily interested in examining participants who remained in the same neighborhood during the entire observation period. By excluding participants who moved, we would yield an association that is easier to conclude that changes in perceived neighborhood cohesion led to changes in SAH and not due to factors associated with moving. Therefore, we included only participants with at least two waves of data who remained in the same neighborhood throughout the observation period. A total of 8963 participants had data across the four waves. We excluded 2694 participants with missing postal codes, 789 participants who changed neighborhoods, and 2882 participants with less than two observations, resulting in a final sample of 2598 participants (see Fig. 1).

### Perceived neighborhood social cohesion

We assess perceived neighborhood social cohesion with a four-item scale based on two widely used instruments [7, 24, 25]. Respondents were asked to what extent they agree or disagree with the following questions about their neighborhood: (a) People in this neighborhood get along with each other pleasantly, (b) People in this neighborhood are willing to help each other (c) I often feel alone in this neighborhood, and (d) I would move out of this neighborhood if I get the chance. The first two items capture perceived collective efficacy, whereas the last two items measure a sense of belonging [25]. Response categories were “strongly agree,” “agree,” “neither agree nor disagree,” “don’t agree,” and “strongly don’t agree,” ranging from one to five. Responses were coded so that higher values indicate a higher perceived neighborhood social cohesion. The individual scores were summed into a new scale with a possible range from 4 to 20. The scale had acceptable internal consistency with Cronbach’s alphas ranging from 0.68 to 0.74 across the four waves.

### Self-assessed health

The outcome variable is SAH, which was assessed by asking the participants the single-question item: How would you rate your health in general? The five response categories ranged from poor, fair, good, very good, to excellent. For the main analysis, the variable was dichotomized into poor health (poor and fair) and good health (good, very good, and excellent), which is consistent with majority of previous research [4].

### Covariates and moderators

We controlled for time-varying and time-invariant variables that could affect the relationship between perceived neighborhood social cohesion and SAH. Age, living arrangement (living with a partner, living without a partner), monthly household income (<€1200, €1200–1800, €1800–2600, >€2600), employment status (employed, unemployed, retired), financial difficulties in the past year (yes, no), the number of people in the household, years of residence in the neighborhood, and homeownership status (homeowner or renter) were included as time-varying covariates. Time-invariant covariates (measured in 2004) were gender (male, female), place of birth (the Netherlands, elsewhere), and highest attained educational level according to the International Standard Classification of Education (low = ISCED 0–2, middle = ISCED 3–4, high = ISCED 5–7). Age, educational level, and gender were also used as moderators.

### Statistical analyses

Missing variables (ranging from homeownership at 0.5% to household income at 12.4%) were assumed to be missing at random and were handled by using the multivariate imputation by chained equations (MICE) package in R [26]. Five imputed datasets were generated and pooled together to report the estimates, expressed in odds ratios (ORs), and their 95% confidence interval (CI). All analyses were conducted in R (version 4.2.2) [27].

Descriptive statistics of the study population were calculated for the 2004 wave of the study. A two-level multilevel logistic random-effects within-between (REWB) model was used to assess the between-individual and within-individual associations of perceived neighborhood social cohesion with poor SAH. Perceived neighborhood social cohesion and the time-varying covariates were decomposed into their individual-specific means (between-individual estimates) and deviations from those individual-specific means (within-individual estimates). Between-individual estimates were calculated as the average individual’s mean value of the variable across all time points, whereas within-individual estimates were calculated as a deviation score of the difference in time point-specific values of each wave and the person-specific mean. The REWB models were adjusted for the time-varying and time-invariant covariates listed above. Moderation by age, educational level, and gender was assessed by including interaction terms between the between-individual and within-individual estimates of perceived neighborhood social cohesion and the respective moderating variables. For the moderation analysis age was dichotomized to adults aged 60 and older and younger adults, which is close to the median age of the study population (57) and the current average retirement age (i.e. 65) in the Netherlands.

Four sensitivity analyses were conducted. First, we examined the associations of perceived neighborhood social cohesion with poor SAH among all participants including those who changed neighborhoods during the observation period, which provides a more comprehensive perspective of the relationship between neighborhood social cohesion and SAH. Second, to detect all within-individual changes in SAH that occurred in our sample, we treated SAH as a continuous variable and estimated the relationship between perceived neighborhood social cohesion with SAH with REWB linear models. Third, we examined the association between perceived neighborhood social cohesion and lagged poor SAH, where health was lagged by one wave. Fourth, we examined the association between SAH and lagged perceived neighborhood social cohesion. The final two analyses assessed potential reverse causality.

## Results

### Descriptive statistics

Table 1 presents a description of the study population in the 2004 wave of the GLOBE study. The study population in 2004 consisted of 2584 respondents, of which 55% were female. The average age was 54.3 years (SD: 12.4). Most respondents had a low educational level (43%) followed by high (29%) and middle (22%). Approximately 14% of the study population reported either fair or poor health. The average perceived neighborhood social cohesion score was 16.0 (SD: 2.4).

**Table 1.** Description of the study population in the 2004 wave of the GLOBE study

Variables	*N* (%) or mean (SD)	Range (min, max)
Total participants	2584	
Outcome		
Self-assessed health		
Good health	2168 (83.9)	
Poor health	356 (13.8)	
Missing (%)	60 (2.3)	
Exposure		
Perceived social cohesion	16.0 (2.4)	(5, 20)
Missing (%)	145 (5.6)	
Time-invariant variables		
Gender (%)		
Male	1149 (44.5)	
Female	1435 (55.5)	
Birthplace (%)		
The Netherlands	2360 (91.3)	
Elsewhere	168 (6.5)	
Missing	56 (2.2)	
Educational levels (%)		
Low	1111 (43.0)	
Middle	590 (22.8)	
High	755 (29.2)	
Missing	128 (5.0)	
Time-varying variables		
Age	54.3 (12.4)	(25, 85)
Living arrangement (%)		
Living with a partner	2086 (80.7)	
Living without a partner	422 (16.3)	
Missing	76 (2.9)	
Employment status (%)		
Employed	1195 (46.3)	
Retired	649 (25.1)	
Unemployed	579 (22.4)	
Missing	321 (12.4)	
Household income (%)		
<€1200	265 (10.3)	
€1200–€1800	572 (22.1)	
€1800–€2600	658 (25.5)	
>€2600	768 (29.7)	
Missing	321 (12.4)	
Financial strain (%)		
No	1808 (69.9)	
Yes	736 (28.5)	
Missing	40 (1.5)	
Years of residence	18.3 (13.0)	(0, 77)
Missing (%)	139 (5.4)	
Homeownership (%)		
Homeowner	1744 (67.5)	
Renter	827 (32.0)	
Missing	13 (0.5)	
Household size (no. of persons)	2.5 (1.3)	(0, 30)
Missing (%)	13 (0.5)	

### Main analysis

Table 2 shows an unadjusted between-individual association, between more perceived neighborhood social cohesion and a lower odds of poor SAH (OR: 0.63; 95% CI: 0.55, 0.71). This relationship was slightly attenuated after adjusting for sociodemographic factors (OR: 0.72; 95% CI: 0.65, 0.80). Within-individual changes in perceived neighborhood social cohesion were not associated with changes in SAH, in both the unadjusted (OR: 0.95; 95% CI: 0.89, 1.02) and adjusted model (OR: 0.96; 95% CI: 0.89, 1.04).

**Table 2.** Between-individual and within-individual associations of perceived neighborhood social cohesion and poor SAH from a two-level multilevel logistic REWB model, and moderation by age, educational level, and gender from participants who remained in the same neighborhood and with at least two waves of data

	Unadjusted model	Adjusted model^a^	Moderation models^a^		
			Age	Educational level	Gender
	OR (95% CI)	OR (95% CI)	OR (95% CI)	OR (95% CI)	OR (95% CI)
Between-individual estimates					
Social cohesion	0.63 (0.55, 0.71)	0.72 (0.65, 0.80)	0.77 (0.66, 0.89)	0.73 (0.63, 0.84)	0.73 (0.63, 0.84)
Old age					
<60			Ref		
≥60			18.96 (0.57, 620.15)		
Educational level					
Low		Ref	Ref	Ref	Ref
Middle		0.65 (0.37, 1.15)	0.57 (0.36, 0.89)	0.34 (0.01, 7.86)	0.58 (0.35, 0.97)
High		0.71 (0.41, 1.22)	0.59 (0.35, 1.01)	1.49 (0.04, 54.86)	0.63 (0.36, 1.09)
Gender					
Male		Ref	Ref	Ref	Ref
Female		0.86 (0.57, 1.31)	0.85 (0.54, 1.34)	0.85 (0.57, 1.27)	1.45 (0.07, 27.73)
*Interaction terms*					
Age × Social Cohesion					
<60			Ref		
≥60			0.88 (0.72, 1.09)		
Educational Level × Social Cohesion					
Low				Ref	
Middle				1.04 (0.85, 1.26)	
High				0.95 (0.75, 1.19)	
Gender × Social Cohesion					
Male					Ref
Female					0.96 (0.80, 1.16)
Within-individual estimates					
Social cohesion	0.95 (0.89, 1.02)	0.96 (0.89, 1.04)	0.93 (0.82, 1.05)	0.94 (0.88, 1.01)	0.93 (0.85, 1.02)
Age		1.08 (1.01, 1.16)		1.09 (1.02, 1.17)	1.11 (1.03, 1.19)
Old age					
<60			Ref		
≥60			0.96 (0.61, 1.49)		
*Interaction terms*					
Age × Social Cohesion					
<60			Ref		
≥60			1.03 (0.87, 1.24)		
Educational Level × Social Cohesion					
Low				Ref	
Middle				1.07 (0.91, 1.25)	
High				0.98 (0.84, 1.14)	
Gender × Social Cohesion					
Male					Ref
Female					1.04 (0.92, 1.18)
AIC^b^	5448	5152	5170	5163	5153
BIC^c^	5497	5380	5413	5419	5395

a Adjusted for age, educational status, gender, birthplace, living arrangement, employment status, household income, financial strain, years of residence, homeownership, and household size.

b Average Akaike information criterion from the five imputed datasets.

c Average Bayesian information criterion from the five imputed datasets.

### Moderation analysis

The results from the moderation analysis indicate that age (OR: 0.88; 95% CI: 0.72, 1.09), educational level (Ref vs. Middle—OR: 1.04; 95% CI: 0.85, 1.26, Ref vs. High—OR: 0.95; 95% CI: 0.75, 1.19), and gender (OR: 0.96; 95% CI: 0.80, 1.16) did not moderate the between-individual association of perceived neighborhood social cohesion and SAH. We also did not find evidence for moderation by age (OR: 1.03; 95% CI: 0.87, 1.24), educational level (Ref vs. Middle—OR: 1.07; 95% CI: 0.91, 1.25, Ref vs. High—OR: 0.98; 95% CI: 0.84, 1.14) or gender (OR: 1.04; 95% CI: 0.92, 1.18) for the within-individual associations.

### Sensitivity analysis

When examining all participants including those who changed neighborhood, we found that perceived neighborhood social cohesion was associated with a lower odds of poor SAH across individuals (OR: 0.74; 95% CI: 0.67, 0.81) after adjusting for sociodemographic factors (Supplementary Table 1), which is in line with the main analysis. The within-individual association between perceived social cohesion and poor SAH was in the same direction as the main analysis, but reached significance (OR: 0.93; 95% CI: 0.88, 0.98) after adjusting for sociodemographic factors.

When treating SAH as continuous variable, we found that higher perceived neighborhood social cohesion across individuals was associated with better SAH after adjusting for sociodemographic factors (β = 0.07; 95% CI: 0.06, 0.08) (Supplementary Table 2), which is consistent with the main analysis. Within-individual changes in perceived neighborhood social cohesion were positively associated with changes in SAH when accounting for sociodemographic factors (β = 0.01; 95% CI: 0.00, 0.02).

Similar to the finding from the main analysis, we observed that higher perceived neighborhood social cohesion was associated with a lower odds of poor health in the subsequent wave across individuals (OR: 0.71; 95% CI: 0.55, 0.93) (Supplementary Table 3). We did not observe within-individual associations between perceived neighborhood social cohesion and the lagged SAH (OR: 0.99; 95% CI: 0.89, 1.11), which is consistent with main analysis.

The results from the final sensitivity analysis indicate that poor SAH was associated with lower perceptions of neighborhood social cohesion in the subsequent wave (β: −0.99; 95% CI: −1.26, −0.71) (Supplementary Table 4). We did not observe within-individual associations of SAH and perceived neighborhood social cohesion in the subsequent wave (β: −0.04; 95% CI: −0.29, 0.21).

## Discussion

The present study used REWB models to assess the between-individual and within-individual associations of perceived neighborhood social cohesion and poor SAH. Our findings suggest that higher perceptions of neighborhood social cohesion are associated with a lower odds of poor SAH. However, we did not find conclusive evidence that increases in perceived neighborhood social cohesion are associated with better SAH within individuals. This analysis also did not find evidence that either of these associations varied by age, educational level, or gender.

Our findings that higher perceptions of neighborhood social cohesion were associated with better SAH across individuals are in line with previous research that examined between-individual associations of perceived neighborhood social cohesion and SAH [4, 28–30] and was confirmed by the two sensitivity analyses that examined all participants and treated SAH as a continuous variable, respectively.

Our findings for the within-individual associations of perceived neighborhood social cohesion and health were not as straightforward. The results from the main analysis, using the dichotomized SAH variable among participants who did not relocate, revealed no clear within-individual association. However, when examining all participants or when using the numeric SAH variable, the results did suggest an association between changes in perceived neighborhood social cohesion and changes in SAH. The lack of association for the main analysis might be attributed to a lack of variation (i.e. a lack of change in binary SAH) in the outcome variable, which is a common limitation of fixed-effects models [31]. Using a dichotomous measure in a within model will only detect large changes or changes at the cut-off point (i.e. between good and fair), but not any changes that occur within the dichotomized categories such as from good to very good. This approach results in excluding a substantial amount of the available variation and a proportion of the sample, which is why the sensitivity analysis was performed. Furthermore, it should be noted that the magnitude of the within-individual associations was minimal and was much smaller than the between-individual associations. On the one hand, this may suggest that the impact of social cohesion on SAH is relatively limited, but on the other hand, this may imply that only drawing on within-individual changes may underestimate the total impact of social cohesion on health. Therefore, future research should leverage different study designs to help establish the true effect of social cohesion on health.

The findings from the lagged analyses were mixed. The between-individual associations showed that perceived neighborhood social cohesion was related to lagged SAH, which is in line with the main findings. However, we observed no within-individual associations in the lagged models. The lengths of the lags varied up to 7 years, which may have been longer than the time it takes for changes in perceived neighborhood social cohesion to impact changes in SAH. In the final sensitivity analysis, we observed no relationships for within-individual associations of SAH and the lagged perceived neighborhood social cohesion. These findings provide some evidence against reverse causality and some validation for the direction of our hypothesis. Due to the lack of robustness of our within-individual associations, we cannot confidently state that these findings support our hypothesis that within-individual changes in perceived neighborhood social cohesion were associated with changes in SAH.

While investigating potential modifiers, we observed no variations in the association between perceived neighborhood social cohesion and health by age, educational level, or gender. Therefore, we cannot confirm that increasing perceptions of neighborhood cohesion is more beneficial for a particular subpopulation. We hypothesized that older adults would benefit more than younger adults since they are more dependent on the social support in the community. However, since aging adults also face physical mobility issues, other neighborhood attributes like quality of the built environment and neighborhood disorder may play a more important role in the health of older adults than perceptions of the neighborhood social environment [32]. The lack of studies that investigates the moderating effect of age on the association between the social environment and health indicates a knowledge gap for future research to address.

The moderating effect of SEP on social cohesion and health is still unclear. Some research support the buffering effects hypothesis, which posits that people from disadvantaged populations will have greater health benefits of social cohesion compared to people from non-disadvantaged populations [17]. However, social cohesion and social capital are broad constructs and encompass numerous dimensions [28]. Our study operationalized collective efficacy and sense of belonging as an index of the social environment. Therefore, it is possible that other dimensions of social cohesion could capture the moderating effect of SEP. Future research could distinguish between the different dimensions of social cohesion and consider their particular relationship with health [28].

Our study observed no differences between men and women. Although gender differences in the association of social cohesion or related social capital variables and health have been observed in prior research [16, 22, 33], it remains unclear which group have more favorable outcomes. Some research suggest that women may benefit more than men from higher levels of social cohesion [16] or area social capital [33] due to differences in the perceptions of the neighborhood, differences in the exposures within the neighborhood, and differences in vulnerabilities to certain aspects of the neighborhood. These findings underscore the interplay of factors explaining why men and women interact with their living environments differently. A plausible explanation for our results is that gender differences could be context-specific, and thus cultural differences in gender norms could influence whether men or women benefit more from changes in the social environment. As a leading country in gender egalitarianism, differences in gender norms might not be prominent in Dutch societies.

We acknowledge that our study also has some limitations. First, the measurements were based on self-reported survey data from the same data source making our results vulnerable to recall and same source biases. It would be methodologically advantageous to use objective measurements from different data sources. Second, dichotomizing SAH resulted in a loss of information and given the limited sample size, the models might suffer from a substantial reduction of statistical power [31]. Similarly, it is also plausible that our models lacked sufficient statistical power to detect the interaction effects.

## Conclusion

Understanding how perceptions of the neighborhood influence health is a top public health priority. This study contributes to the literature by simultaneously examining the between-individual and within-individual associations of perceived neighborhood social cohesion and SAH. Our main findings provide further evidence for the relationship between perceived neighborhood social cohesion and SAH and provide some evidence that increasing perceived neighborhood social cohesion may impact residents’ health over time. With these new insights, policymakers can design interventions that improve health and reduce health disparities.

## Supplementary Material

ckae168_Supplementary_Data

## Data Availability

The data underlying this article will be shared upon reasonable request to the corresponding author.
Key points Higher perceived neighborhood social cohesion is associated with better self-assessed health. The association between within-individual changes in perceived neighborhood social cohesion and within-individual changes in health is inconclusive. The association between perceived neighborhood social cohesion and self-assessed health does not vary by age, educational level, or gender.
