# Supplementary material for: Changes in perceived neighborhood social cohesion and self-assessed health: 17-year follow-up of the Dutch GLOBE study
Source: Eur J Public Health. 2024 Nov 1;34(6):1079–85. doi: 10.1093/eurpub/ckae168 (PMC11631403; doi:10.1093/eurpub/ckae168)
Supplement: ckae168_Supplementary_Data [file ckae168_supplementary_data.docx]

Supplementary Table 1. Between-individual and within-individual associations of perceived neighborhood social cohesion and poor SAH with covariate coefficients from a two-level multilevel logistic REWB model, and moderation by age, gender and educational level from all participants

|  | Unadjusted model | Adjusted model | Moderation models | | |
| --- | --- | --- | --- | --- | --- |
|  |  |  | Age | Educational level | Gender |
|  | OR (95%CI) | OR (95%CI) | OR (95%CI) | OR (95%CI) | OR (95%CI) |
| **Between-individual estimates** |  |  |  |  |  |
| Social cohesion | 0.79 (0.74, 0.84) | 0.74 (0.67, 0.81) | 0.85 (0.77, 0.94) | 0.84 (0.65, 1.09) | 0.80 (0.67, 0.95) |
| Age |  | 1.07 (1.04, 1.10) |  | 1.05 (1.00, 1.11) | 1.07 (1.04, 1.09) |
| Old age |  |  |  |  |  |
| < 60 |  |  | Ref |  |  |
| >= 60 |  |  | 60.00 (0.37, 9616) |  |  |
| Educational level |  |  |  |  |  |
| Low |  | Ref | Ref | Ref | Ref |
| Middle |  | 0.63 (0.30, 1.30) | 0.53 (0.18, 1.56) | 6.95 (0.43, 112.29) | 0.51 (0.20, 1.30) |
| High |  | 0.51 (0.22, 1.15) | 0.45 (0.17, 1.14) | 2.26 (0.05, 101.50) | 0.44 (0.23, 0.86) |
| Gender |  |  |  |  |  |
| Male |  | Ref | Ref | Ref | Ref |
| Female |  | 0.88 (0.47, 1.65) | 0.73 (0.35, 1.52) | 0.93 (0.37, 2.31) | 2.18 (0.30, 15.67) |
| Birthplace |  |  |  |  |  |
| Elsewhere |  | Ref | Ref | Ref | Ref |
| Netherlands |  | 0.57 (0.27, 1.18) | 0.56 (0.31, 1.01) | 0.54 (0.23, 1.23) | 0.46 (0.28, 0.76) |
| Living arrangements |  |  |  |  |  |
| Without a partner |  | Ref | Ref | Ref | Ref |
| With a partner |  | 1.10 (0.48, 2.49) | 1.23 (0.57, 2.68) | 0.88 (0.35, 2.19) | 0.91 (0.55, 1.50) |
| Employment |  |  |  |  |  |
| Employed |  | Ref | Ref | Ref | Ref |
| Retired |  | 1.35 (0.49, 3.70) | 4.12 (1.49, 11.35) | 1.90 (0.50, 7.21) | 1.36 (0.32, 5.75) |
| Unemployed |  | 6.04 (2.18, 16.69) | 10.20 (5.35 19.44) | 6.26 (1.44, 27.12) | 5.78 (2.78, 12.00) |
| Household income |  |  |  |  |  |
| <€1200 |  | Ref | Ref | Ref | Ref |
| €1200–€1800 |  | 0.19 (0.06, 0.59) | 0.28 (0.04, 1.92) | 0.36 (0.05, 2.42) | 0.42 (0.05, 3.15) |
| €1800–€2600 |  | 0.18 (0.03, 0.85) | 0.27 (0.04, 1.56) | 0.28 (0.08, 1.01) | 0.31 (0.09, 1.06) |
| >€2600 |  | 0.12 (0.01, 0.86) | 0.14 (0.02, 0.91) | 0.21 (0.04, 1.13) | 0.24 (0.05, 1.06) |
| Financial strain |  |  |  |  |  |
| No |  | Ref | Ref | Ref | Ref |
| Yes |  | 4.63 (1.83, 11.68) | 4.08 (2.16, 7.72) | 4.06 (2.39, 6.90) | 4.56 (2.09, 9.96) |
| Years of residence |  | 0.97 (0.95, 1.00) | 0.98 (0.97, 1.00) | 0.98 (0.95, 1.00) | 0.97 (0.94, 1.01) |
| Home ownership |  |  |  |  |  |
| Renter |  | Ref | Ref | Ref | Ref |
| Home owner |  | 0.52 (0.28, 0.98) | 0.55 (0.30, 0.98) | 0.54 (0.23, 1.30) | 0.55 (0.33, 0.93) |
| Household size |  | 0.89 (0.79, 1.00) | 0.87 (0.71, 1.06) | 0.92 (0.83, 1.02) | 0.89 (0.80, 0.99) |
| *Interaction terms* |  |  |  |  |  |
| Age x Social cohesion |  |  |  |  |  |
| < 60 |  |  | Ref |  |  |
| >= 60 |  |  | 0.81 (0.59, 1.10) |  |  |
| Educational level x Social cohesion |  |  |  |  |  |
| Low |  |  |  | Ref |  |
| Middle |  |  |  | 0.86 (0.71, 1.04) |  |
| High |  |  |  | 0.91 (0.71, 1.16) |  |
| Gender x Social cohesion |  |  |  |  |  |
| Male |  |  |  |  | Ref |
| Female |  |  |  |  | 0.94 (0.83, 1.07) |
| **Within-individual estimates** |  |  |  |  |  |
| Social cohesion | 0.92 (0.86, 1.00) | 0.93 (0.88, 0.98) | 0.90 (0.70, 1.15) | 0.91 (0.84, 0.99) | 0.91 (0.75, 1.12) |
| Age |  | 1.12 (1.07, 1.17) |  | 1.10 (1.02, 1.19) | 1.11 (1.04, 1.18) |
| Older age |  |  |  |  |  |
| < 60 |  |  | Ref |  |  |
| >= 60 |  |  | 0.76 (0.43, 1.35) |  |  |
| Living arrangements |  |  |  |  |  |
| Without a partner |  | Ref | Ref | Ref | Ref |
| With a partner |  | 0.94 (0.34, 2.53) | 0.75 (0.43, 1.28) | 0.93 (0.50, 1.76) | 0.83 (0.34, 2.02) |
| Employment |  |  |  |  |  |
| Employed |  | Ref | Ref | Ref | Ref |
| Retired |  | 0.92 (0.52, 1.60) | 1.22 (0.45, 3.32) | 1.13 (0.38, 3.37) | 1.00 (0.45, 2.18) |
| Unemployed |  | 2.55 (1.53, 4.23) | 3.46 (1.04, 11.45) | 2.58 (1.19, 5.56) | 2.31 (0.55, 9.69) |
| Household income |  |  |  |  |  |
| <€1200 |  | Ref | Ref | Ref | Ref |
| €1200–€1800 |  | 0.93 (0.57, 1.53) | 0.89 (0.47, 1.66) | 1.15 (0.55, 2.40) | 1.35 (0.86, 2.14) |
| €1800–€2600 |  | 0.97 (0.53, 1.77) | 0.93 (0.36, 2.39) | 1.14 (0.36, 3.55) | 1.55 (0.58, 4.16) |
| >€2600 |  | 0.94 (0.45, 1.94) | 0.90 (0.21, 3.81) | 1.08 (0.19, 5.91) | 1.49 (0.50, 4.41) |
| Financial strain |  |  |  |  |  |
| No |  | Ref | Ref | Ref | Ref |
| Yes |  | 1.85 (1.16, 2.94) | 1.57 (0.84, 2.93) | 1.62 (0.81, 3.22) | 1.73 (0.91, 3.31) |
| Years of residence |  | 0.99 (0.97, 1.01) | 0.99 (0.96, 1.02) | 0.99 (0.96, 1.01) | 0.99 (0.98, 1.01) |
| Home ownership |  |  |  |  |  |
| Renter |  | Ref | Ref | Ref | Ref |
| Home owner |  | 0.59 (0.16, 2.21) | 0.59 (0.06, 5.52) | 0.71 (0.21, 2.40) | 0.56 (0.28, 1.11) |
| Household size |  | 0.97 (0.88, 1.06) | 0.95 (0.84, 1.07) | 0.96 (0.85, 1.08) | 0.95 (0.87, 1.04) |
| *Interaction terms* |  |  |  |  |  |
| Age x Social cohesion |  |  |  |  |  |
| < 60 |  |  | Ref |  |  |
| >= 60 |  |  | 1.05 (0.72, 1.53) |  |  |
| Educational level x Social cohesion |  |  |  |  |  |
| Low |  |  |  | Ref |  |
| Middle |  |  |  | 1.09 (0.94, 1.26) |  |
| High |  |  |  | 0.99 (0.83, 1.18) |  |
| Gender x Social cohesion |  |  |  |  |  |
| Male |  |  |  |  | Ref |
| Female |  |  |  |  | 1.04 (0.80, 1.35) |
| AIC ^a^ | 9301 | 8942 | 9063 | 9020 | 8985 |
| BIC ^b^ | 9353 | 9187 | 9324 | 9295 | 9246 |

^a^ average Akaike information criterion from the five imputed datasets,

^b^ average Bayesian information criterion from the five imputed datasets

Supplementary Table 2. Between-individual and within-individual associations of perceived neighborhood social cohesion and SAH with covariate coefficients from a two-level multilevel linear REWB model, and moderation by age, educational level, and gender from participants who remained in the same neighborhood and with at least two waves of data

|  | Unadjusted model | Adjusted model | Moderation models | | |
| --- | --- | --- | --- | --- | --- |
|  |  |  | Age | Educational level | Gender |
|  | β (95%CI) | β (95%CI) | β (95%CI) | β (95%CI) | β (95%CI) |
| **Between-individual estimates** |  |  |  |  |  |
| Social cohesion | 0.09 (0.08; 0.11) | 0.07 (0.06; 0.08) | 0.06 (0.04; 0.08) | 0.06 (0.04; 0.08) | 0.07 (0.05; 0.08) |
| Age |  | -0.01 (-0.01; -0.00) |  | -0.01 (-0.01; -0.00) | -0.01 (-0.01; -0.01) |
| Old age |  |  |  |  |  |
| < 60 |  |  | Ref |  |  |
| >= 60 |  |  | -0.19 (-0.66; 0.26) |  |  |
| Educational level |  |  |  |  |  |
| Low |  | Ref | Ref | Ref | Ref |
| Middle |  | 0.10 (0.03; 0.17) | 0.11 (0.05; 0.18) | 0.10 (-0.36; 0.58) | 0.10 (0.03; 0.17) |
| High |  | 0.11 (0.04; 0.19) | 0.13 (0.05; 0.20) | -0.31 (-0.80; 0.17) | 0.11 (0.04; 0.19) |
| Gender |  |  |  |  |  |
| Male |  | Ref | Ref | Ref | Ref |
| Female |  | -0.01 (-0.06; 0.04) | -0.01 (-0.06; 0.04) | -0.01 (-0.06; 0.04) | -0.08 (-0.47; 0.30) |
| Birthplace |  |  |  |  |  |
| Elsewhere |  | Ref | Ref | Ref | Ref |
| Netherlands |  | 0.07 (-0.03; 0.17) | 0.07 (-0.03; 0.17) | 0.07 (-0.02; 0.18) | 0.07 (-0.03; 0.17) |
| Living arrangements |  |  |  |  |  |
| Without a partner |  | Ref | Ref | Ref | Ref |
| With a partner |  | -0.06 (-0.15; 0.01) | -0.07 (-0.16; 0.01) | -0.07 (-0.15; 0.01) | -0.06 (-0.15; 0.01) |
| Employment |  |  |  |  |  |
| Employed |  | Ref | Ref | Ref | Ref |
| Retired |  | -0.10 (-0.22; 0.00) | -0.24 (-0.38; -0.11) | -0.10 (-0.22; 0.01) | -0.10 (-0.22; 0.01) |
| Unemployed |  | -0.35 (-0.46; -0.24) | -0.41 (-0.53; -0.30) | -0.35 (-0.45; -0.24) | -0.35 (-0.46; -0.24) |
| Household income |  |  |  |  |  |
| <€1200 |  | Ref | Ref | Ref | Ref |
| €1200–€1800 |  | 0.12 (-0.01; 0.26) | 0.13 (-0.00; 0.27) | 0.12 (-0.00; 0.26) | 0.12 (-0.01; 0.26) |
| €1800–€2600 |  | 0.12 (-0.00; 0.26) | 0.13 (-0.00; 0.26) | 0.13 (-0.00; 0.27) | 0.12 (-0.00; 0.26) |
| >€2600 |  | 0.28 (0.13; 0.44) | 0.29 (0.13; 0.44) | 0.28 (0.13; 0.44) | 0.28 (0.13; 0.43) |
| Financial strain |  |  |  |  |  |
| No |  | Ref | Ref | Ref | Ref |
| Yes |  | -0.25 (-0.33; -0.17) | -0.25 (-0.33; -0.16) | -0.25 (-0.33; -0.17) | -0.25 (-0.33; -0.17) |
| Years of residence |  | 0.00 (-0.00; 0.00) | 0.00 (-0.00; 0.00) | 0.00 (-0.00; 0.00) | 0.00 (-0.00; 0.00) |
| Home ownership |  |  |  |  |  |
| Renter |  | Ref | Ref | Ref | Ref |
| Home owner |  | -0.00 (-0.07; 0.06) | 0.00 (-0.06; 0.06) | -0.00 (-0.07; 0.06) | -0.00 (-0.07; 0.06) |
| Household size |  | 0.01 (-0.00; 0.02) | 0.01 (0.00; 0.02) | 0.01 (-0.00; 0.02) | 0.01 (-0.00; 0.02) |
| *Interaction terms* |  |  |  |  |  |
| Age x Social cohesion |  |  |  |  |  |
| < 60 |  |  | Ref |  |  |
| >= 60 |  |  | 0.00 (-0.02; 0.03) |  |  |
| Educational level x Social cohesion |  |  |  |  |  |
| Low |  |  |  | Ref |  |
| Middle |  |  |  | -0.00 (-0.02; 0.02) |  |
| High |  |  |  | 0.02 (-0.00; 0.05) |  |
| Gender x Social cohesion |  |  |  |  |  |
| Male |  |  |  |  | Ref |
| Female |  |  |  |  | 0.00 (-0.01; 0.02) |
| **Within-individual estimates** |  |  |  |  |  |
| Social cohesion | 0.01 (0.00; 0.02) | 0.01 (0.00; 0.02) | 0.01 (-0.00; 0.02) | 0.01 (0.00; 0.02) | 0.01 (-0.00; 0.02) |
| Age |  | -0.01 (-0.02; -0.00) |  | -0.01 (-0.02; -0.00) | -0.01 (-0.02; -0.00) |
| Old age |  |  |  |  |  |
| < 60 |  |  | Ref |  |  |
| >= 60 |  |  | 0.02 (-0.03; 0.08) |  |  |
| Living arrangements |  |  |  |  |  |
| Without a partner |  | Ref | Ref | Ref | Ref |
| With a partner |  | -0.02 (-0.10; 0.05) | -0.02 (-0.10; 0.05) | -0.02 (-0.10; 0.05) | -0.02 (-0.10; 0.05) |
| Employment |  |  |  |  |  |
| Employed |  | Ref | Ref | Ref | Ref |
| Retired |  | 0.01 (-0.05; 0.08) | 0.00 (-0.07; 0.07) | 0.01 (-0.05; 0.08) | 0.01 (-0.05; 0.08) |
| Unemployed |  | -0.11 (-0.19; -0.03) | -0.12 (-0.20; -0.04) | -0.11 (-0.19; -0.03) | -0.11 (-0.19; -0.03) |
| Household income |  |  |  |  |  |
| <€1200 |  | Ref | Ref | Ref | Ref |
| €1200–€1800 |  | -0.02 (-0.11; 0.05) | -0.03 (-0.11; 0.05) | -0.02 (-0.11; 0.05) | -0.02 (-0.11; 0.05) |
| €1800–€2600 |  | -0.01 (-0.12; 0.09) | -0.01 (-0.12; 0.08) | -0.01 (-0.12; 0.09) | -0.01 (-0.12; 0.09) |
| >€2600 |  | 0.01 (-0.11; 0.13) | 0.00 (-0.11; 0.12) | 0.01 (-0.11; 0.13) | 0.01 (-0.11; 0.13) |
| Financial strain |  |  |  |  |  |
| No |  | Ref | Ref | Ref | Ref |
| Yes |  | -0.03 (-0.09; 0.01) | -0.03 (-0.09; 0.01) | -0.03 (-0.09; 0.01) | -0.03 (-0.09; 0.01) |
| Years of residence |  | -0.00 (-0.00; 000) | -0.00 (-0.00; 0.00) | -0.00 (-0.00; 0.00) | -0.00 (0.00; 0.00) |
| Home ownership |  |  |  |  |  |
| Renter |  | Ref | Ref | Ref | Ref |
| Home owner |  | 0.04 (-0.13; 0.21) | 0.04 (-0.13; 0.21) | 0.04 (-0.13; 0.21) | 0.04 (-0.13; 0.21) |
| Household size |  | 0.00 (-0.00; 0.01) | 0.00 (-0.00; 0.01) | 0.00 (-0.00; 0.01) | 0.00 (-0.00; 0.01) |
| *Interaction terms* |  |  |  |  |  |
| Age x Social cohesion |  |  |  |  |  |
| < 60 |  |  | Ref |  |  |
| >= 60 |  |  | 0.00 (-0.01; 0.02) |  |  |
| Educational level x Social cohesion |  |  |  |  |  |
| Low |  |  |  | Ref |  |
| Middle |  |  |  | -0.00 (-0.02; 0.02) |  |
| High |  |  |  | -0.00 (-0.02; 0.01) |  |
| Gender x Social cohesion |  |  |  |  |  |
| Male |  |  |  |  | Ref |
| Female |  |  |  |  | 0.00 (-0.01; 0.02) |
| AIC ^a^ | 16134.96 | 15727.01 | 15768.42 | 15758.98 | 15744.50 |
| BIC ^b^ | 16190.30 | 15962.21 | 16017.45 | 16021.84 | 15993.53 |

^a^ average Akaike information criterion from the five imputed datasets,

^b^ average Bayesian information criterion from the five imputed datasets

Supplementary Table 3. Between-individual and within-individual associations of perceived neighborhood social cohesion and the lagged poor SAH from a two-level multilevel logistic REWB model

|  | Unadjusted Model | Adjusted Model ^c^ |
| --- | --- | --- |
|  | OR (95%CI) | OR (95%CI) |
| **Between-individual estimates** |  |  |
| Social cohesion | 0.73 (0.64, 0.83) | 0.71 (0.55, 0.93) |
|  |  |  |
| **Within-individual estimates** |  |  |
| Social cohesion | 1.04 (0.64, 1.15) | 0.99 (0.89, 1.11) |
|  |  |  |
| AIC ^a^ | 4674 | 4713 |
| BIC ^b^ | 4714 | 4929 |

^a^ average Akaike information criterion from the five imputed datasets,

^b^ average Bayesian information criterion from the five imputed datasets,

^c^ adjusted for age, educational status, gender, birthplace, living arrangements, employment status, household income, financial strain, years of residence, home ownership, household size

Supplementary Table 4. Between-individual and within-individual associations of SAH and lagged perceived neighborhood social cohesion from a two-level multilevel linear REWB model

|  | Unadjusted Model | Adjusted Model ^c^ |
| --- | --- | --- |
|  | β (95%CI) | β (95%CI) |
| **Between-individual estimates** |  |  |
| Self-assessed health |  |  |
| Good health | Ref | Ref |
| Poor health | -1.46 (-1.71, -1.20) | -0.99 (-1.26, -0.71) |
|  |  |  |
| **Within-individual estimates** |  |  |
| Self-assessed health |  |  |
| Good health | Ref | Ref |
| Poor health | -0.07 (-0.32, 0.17) | -0.04 (-0.29, 0.21) |
|  |  |  |
| AIC ^a^ | 28824 | 4713 |
| BIC ^b^ | 28871 | 4929 |

^a^ average Akaike information criterion from the five imputed datasets,

^b^ average Bayesian information criterion from the five imputed datasets,

^c^ adjusted for age, educational status, gender, birthplace, living arrangements, employment status, household income, financial strain, years of residence, home ownership, household size

Supplementary Table 5. Between-individual and within-individual associations of perceived neighborhood social cohesion and poor SAH with covariate coefficients from a two-level multilevel logistic REWB model, and moderation by age, educational level, and gender from participants who remained in the same neighborhood and with at least two waves of data

|  | Unadjusted model | Adjusted model | Moderation models | | |
| --- | --- | --- | --- | --- | --- |
|  |  |  | Age | Educational level | Gender |
|  | OR (95%CI) | OR (95%CI) | OR (95%CI) | OR (95%CI) | OR (95%CI) |
| **Between-individual estimates** |  |  |  |  |  |
| Social cohesion | 0.63 (0.55, 0.71) | 0.72 (0.65, 0.80) | 0.77 (0.66, 0.89) | 0.73 (0.63, 0.84) | 0.73 (0.63, 0.84) |
| Age |  | 1.06 (1.03, 1.09) |  | 1.05 (1.02, 1.08) | 1.05 (1.02, 1.09) |
| Old age |  |  |  |  |  |
| < 60 |  |  | Ref |  |  |
| >= 60 |  |  | 18.96 (0.57, 620.15) |  |  |
| Educational level |  |  |  |  |  |
| Low |  | Ref | Ref | Ref | Ref |
| Middle |  | 0.65 (0.37, 1.15) | 0.57 (0.36, 0.89) | 0.34 (0.01, 7.86) | 0.58 (0.35, 0.97) |
| High |  | 0.71 (0.41, 1.22) | 0.59 (0.35, 1.01) | 1.49 (0.04, 54.86) | 0.63 (0.36, 1.09) |
| Gender |  |  |  |  |  |
| Male |  | Ref | Ref | Ref | Ref |
| Female |  | 0.86 (0.57, 1.31) | 0.85 (0.54, 1.34) | 0.85 (0.57, 1.27) | 1.45 (0.07, 27.73) |
| Birthplace |  |  |  |  |  |
| Elsewhere |  | Ref | Ref | Ref | Ref |
| Netherlands |  | 0.50 (0.26, 0.97) | 0.49 (0.25, 0.95) | 0.50 (0.20, 1.25) | 0.49 (0.26, 0.94) |
| Living arrangements |  |  |  |  |  |
| Without a partner |  | Ref | Ref | Ref | Ref |
| With a partner |  | 1.10 (0.48, 2.49) | 1.13 (0.50, 2.54) | 1.17 (0.62, 2.19) | 1.14 (0.57, 2.29) |
| Employment |  |  |  |  |  |
| Employed |  | Ref | Ref | Ref | Ref |
| Retired |  | 1.47 (0.56, 3.88) | 2.21 (0.87, 5.58) | 1.83 (0.79, 4.24) | 1.55 (0.62, 3.85) |
| Unemployed |  | 6.82 (3.26, 14.24) | 7.93 (3.84, 16.38) | 7.44 (3.69, 14.99) | 7.02 (3.22, 15.32) |
| Household income |  |  |  |  |  |
| <€1200 |  | Ref | Ref | Ref | Ref |
| €1200–€1800 |  | 0.34 (0.15, 0.80) | 0.33 (0.13, 0.81) | 0.43 (0.16, 1.11) | 0.35 (0.13, 0.89) |
| €1800–€2600 |  | 0.28 (0.12, 0.67) | 0.27 (0.10, 0.70) | 0.33 (0.13, 0.80) | 0.27 (0.11, 0.70) |
| >€2600 |  | 0.12 (0.04, 0.36) | 0.13 (0.04, 0.40) | 0.18 (0.06, 0.49) | 0.14 (0.05, 0.42) |
| Financial strain |  |  |  |  |  |
| No |  | Ref | Ref | Ref | Ref |
| Yes |  | 2.57 (1.40, 4.73) | 2.60 (1.40, 4.81) | 2.74 (1.60, 4.69) | 2.63 (1.50, 4.62) |
| Years of residence |  | 0.97 (0.95, 0.99) | 0.98 (0.96, 0.99) | 0.98 (0.96, 0.99) | 0.98 (0.96, 0.99) |
| Home ownership |  |  |  |  |  |
| Renter |  | Ref | Ref | Ref | Ref |
| Home owner |  | 0.68 (0.42, 1.09) | 0.68 (0.45, 1.02) | 0.66 (0.39, 1.11) | 0.67 (0.42, 1.06) |
| Household size |  | 0.98 (0.86, 1.11) | 0.95 (0.85, 1.06) | 0.98 (0.89, 1.07) | 0.99 (0.87, 1.12) |
| *Interaction terms* |  |  |  |  |  |
| Age x Social cohesion |  |  |  |  |  |
| < 60 |  |  | Ref |  |  |
| >= 60 |  |  | 0.88 (0.72, 1.09) |  |  |
| Educational level x Social cohesion |  |  |  |  |  |
| Low |  |  |  | Ref |  |
| Middle |  |  |  | 1.04 (0.85, 1.26) |  |
| High |  |  |  | 0.95 (0.75, 1.19) |  |
| Gender x Social cohesion |  |  |  |  |  |
| Male |  |  |  |  | Ref |
| Female |  |  |  |  | 0.96 (0.80, 1.16) |
| **Within-individual estimates** |  |  |  |  |  |
| Social cohesion | 0.95 (0.89, 1.02) | 0.96 (0.89, 1.04) | 0.93 (0.82, 1.05) | 0.94 (0.88, 1.01) | 0.93 (0.85, 1.02) |
| Age |  | 1.08 (1.01, 1.16) |  | 1.09 (1.02, 1.17) | 1.11 (1.03, 1.19) |
| Old age |  |  |  |  |  |
| < 60 |  |  | Ref |  |  |
| >= 60 |  |  | 0.96 (0.61, 1.49) |  |  |
| Living arrangements |  |  |  |  |  |
| Without a partner |  | Ref | Ref | Ref | Ref |
| With a partner |  | 1.10 (0.59, 2.05) | 0.91 (0.46, 1.79) | 0.96 (0.49, 1.87) | 1.11 (0.61, 2.01) |
| Employment |  |  |  |  |  |
| Employed |  | Ref | Ref | Ref | Ref |
| Retired |  | 1.03 (0.58, 1.85) | 1.09 (0.56, 2.10) | 0.98 (0.61, 1.60) | 1.05 (0.61, 1.78) |
| Unemployed |  | 2.18 (1.31, 3.62) | 2.37 (1.22, 4.59) | 1.98 (1.17, 3.36) | 2.17 (1.25, 3.75) |
| Household income |  |  |  |  |  |
| <€1200 |  | Ref | Ref | Ref | Ref |
| €1200–€1800 |  | 1.11 (0.64, 1.94) | 1.27 (0.64, 2.52) | 1.07 (0.62, 1.83) | 1.25 (0.71, 2.20) |
| €1800–€2600 |  | 1.07 (0.54, 2.10) | 1.22 (0.66, 2.23) | 1.00 (0.55, 1.79) | 1.19 (0.65, 2.16) |
| >€2600 |  | 0.95 (0.45, 2.02) | 1.12 (0.48, 2.63) | 0.90 (0.43, 1.87) | 1.06 (0.51, 2.22) |
| Financial strain |  |  |  |  |  |
| No |  | Ref | Ref | Ref | Ref |
| Yes |  | 1.61 (1.11, 2.33) | 1.49 (0.91, 2.45) | 1.45 (1.00, 2.09) | 1.51 (1.05, 2.17) |
| Years of residence |  | 1.01 (0.99, 1.03) | 1.01 (1.00, 1.03) | 1.01 (0.99, 1.02) | 1.01 (0.99, 1.02) |
| Home ownership |  |  |  |  |  |
| Renter |  | Ref | Ref | Ref | Ref |
| Home owner |  | 1.28 (0.41, 3.96) | 1.65 (0.29, 9.38) | 1.27 (0.32, 4.96) | 1.50 (0.44, 5.09) |
| Household size |  | 0.98 (0.92, 1.05) | 0.98 (0.91, 1.05) | 0.98 (0.91, 1.06) | 0.97 (0.91, 1.05) |
| *Interaction terms* |  |  |  |  |  |
| Age x Social cohesion |  |  |  |  |  |
| < 60 |  |  | Ref |  |  |
| >= 60 |  |  | 1.03 (0.87, 1.24) |  |  |
| Educational level x Social cohesion |  |  |  |  |  |
| Low |  |  |  | Ref |  |
| Middle |  |  |  | 1.07 (0.91, 1.25) |  |
| High |  |  |  | 0.98 (0.84, 1.14) |  |
| Gender x Social cohesion |  |  |  |  |  |
| Male |  |  |  |  | Ref |
| Female |  |  |  |  | 1.04 (0.92, 1.18) |
| AIC ^a^ | 5448 | 5152 | 5170 | 5163 | 5153 |
| BIC ^b^ | 5497 | 5380 | 5413 | 5419 | 5395 |

^a^ average Akaike information criterion from the five imputed datasets,

^b^ average Bayesian information criterion from the five imputed datasets
